# Tight-Lacing, Cycling, and Nephroptosis

**Published:** 1901-12-21

**Authors:** 


### TIGHT-LACING, CYCLING, AND NEPHROPTOSIS.

Dr. Alex. Macgregor,[1] writing on floating kidney, points out how much more frequently this condition is met with in women than in men, which, he says, puts it beyond doubt that there must be a cause at work in women which rarely or never acts in the male. This, he thinks, is tight-lacing *plus* muscular effort, and he gives examples to show that cycling may in this way give rise to displacement of the kidney. One patient, whose symptoms were such as to suggest malignant disease of the stomach, dated the onset of her illness from a 50-mile bicycle ride, and another patient, of excellent physique, began to suffer from the characteristic symptoms immediately after a 12-mile ride over a hilly road against time. Two other female patients gave falls in the hunting field as the probable cause of their complaints. Dr. Macgregor shows how often floating kidney may be a cause of painful dyspepsia ; or if he does not quite do this, at least he shows how these dyspeptic troubles may be caused by that general relaxation of the attachments of the abdominal organs of which in some cases nephroptosis is one manifestation. The point of interest, however, to which we draw attention, is the suggestion that when dyspeptic symptoms arise in a tight-lacer, and when they can be traced back to some over-exertion, we should look out for floating kidney. In regard to treatment, Dr. Macgregor's experience seems to have been principally confined to the use of pads and medicinal measures, but many cases occur in which nothing short of operation seems to do any permanent good.

[1] Lancet, Dec. 14.

